# A Facile One-Step Synthesis of Polystyrene/Cellulose (PS@MFC) Biocomposites for the Preparation of Hybrid Water-Absorbing Sponge Materials

**DOI:** 10.3390/polym15214328

**Published:** 2023-11-05

**Authors:** Kirill Cherednichenko, Kristina Bardina, Alexandra Vishnevich, Mariia Gablina, Anastasia Gataulina, Yaroslav Nikolaev, Pavel Gushchin, Evgenii Ivanov, Dmitry Kopitsyn, Vladimir Vinokurov

**Affiliations:** Department of Physical and Colloidal Chemistry, Faculty of Chemical and Environmental Engineering, National University of Oil and Gas (Gubkin University), 65 Leninsky Prospekt, 119991 Moscow, Russia; suhomlina.k@gubkin.ru (K.B.); gataulina.a@gmail.com (A.G.); ivanov166@list.ru (E.I.);

**Keywords:** cellulose microfibrils (MFC), Pickering emulsion, polystyrene (PS), hybrid sponge material, water desalination

## Abstract

The elaboration of a low-cost and effective approach to synthesize hybrid composite materials based on the conventional thermoplastics and natural biopolymers is a sustainable alternative to the production of “traditional” plastics. Cellulose is one of the most abundant biopolymers. Its fibrils possess outstanding mechanical characteristics and, hence, attract considerable interest of researchers during recent decades. However, modification of the hydrophobic polymer matrix by cellulose fibrils is significantly complicated by the hydrophilic nature of the latter. In this study, we propose an effective and low-cost approach to the synthesis of polystyrene at the cellulose microfibrils composite material via the emulsion polymerization method. The obtained fibrous composite was comprehensively analyzed with FTIR spectroscopy, SEM, TGA, and DSC, and was further employed to produce sponge hybrid materials. We investigated the influence of the cellulose/polystyrene ratio on the density, porosity, pore volume, and water uptake of the obtained sponge materials. The sample containing 70 wt.% of cellulose demonstrated the best water absorption properties while preserving its shape, even after 24 h of floating on water. The produced sponge materials might be employed as sorption materials for the purification and desalination of waters of various origins, filtration, and collection of undesirable elements under specific industrial or natural conditions.

## 1. Introduction

The proper management and recycling of plastic materials are serious problems in today’s society. Despite the advantageous features of plastic materials, such as their long functional life, high chemical stability, diversity of mechanical/physical properties, ease of production, and low production costs, plastic pollution is currently considered to be one of the most important environmental threats that can lead to a serious energy crisis [1,2]. Plastic is a dangerous, poorly reversible pollutant due to its extremely slow natural decomposition processes [3].

The creation of eco-friendly polymeric materials is therefore a sustainable alternative to the use of conventional plastics, aiming to develop the safest and most environmentally beneficial strategies for the production of a wide range of materials. Besides the lower environmental impact of such materials, they must be able to fully reproduce the properties of conventional plastics [4]. In spite of the growing industrial demand for new eco-friendly polymeric materials during recent decades and hence, the increasing number of studies that have been performed in this field, conventional polymers (produced from hydrocarbon raw materials) still remain the most common in the global market, owing to the widespread effective and low-cost production technologies as compared to the “green” ones. In this regard, the elaboration of hybrid materials based on thermoplastic synthetic polymers and low-cost biopolymers might become an alternative approach to keep biocomposite production costs low while reducing plastic pollution. Such abundant biopolymers as cellulose, chitin, chitosan, and starch can be integrated into a polymer matrix, preserving its mechanical/physical properties while reducing the environmental impact [5,6,7,8].

Cellulose is one of the most accessible and renewable natural polymers. The distinct advantages of cellulose are its excellent biodegradability and biocompatibility, as well as its prominent mechanical properties. The growing use of cellulose-based composites has been observed in agriculture, construction, medicine, food, etc. The production of packaging materials [9], the creation of disposable biodegradable tableware [10], the development of woven and nonwoven fiber materials [11,12], the creation of energy storage devices [13,14], and the preparation of composite materials for biomedical applications [15,16,17] have become widespread. Cellulose-based biocomposites have recently been of particular interest for the elaboration of porous sorption materials suitable for wastewater treatment [18,19,20], seawater desalination [21,22,23,24], air filtration [25], adsorption of various dyes [20], and materials designed to collect oil spills from offshore fields [26].

The properties of the biocomposites are largely determined by the amount, orientation, size, and aspect ratio of cellulose fibers in their composition. Moreover, the adhesion of cellulose to the matrix is of significant importance, since the complexity of combining hydrophobic polymers with hydrophilic cellulose fibers is a key problem when choosing a method for biocomposite synthesis. One of the possible approaches to enhance cellulose adhesion to polymers is modification of fibrils surface. Thus, for instance, Kim Sung-Hoon et al. improved the compatibility of polypropylene with cellulose microfibrils via its surface silylation [27]. However, modification of the hydroxyl groups on the cellulose surface can significantly impact hydrogen bonding with the polymer matrix and, hence, the mechanical properties of the final composite. An alternative way of obtaining biocomposite materials is the Pickering emulsion approach, which allows us to reach interfacial compatibility of the neat cellulose with the polymer matrix [28,29,30,31]. For example, nanofibrillated cellulose (NFC) [28] and microfibrillated cellulose (MFC) [29] were used to stabilize polystyrene (PS) prepared via the Pickering emulsion polymerization process. Such a production of cellulose-based composites can be considered a two-step procedure, since a styrene polymerization was performed prior to the cellulose addition. Despite a rather complicated synthesis procedure, the obtained composites contained comparatively high amounts of cellulose: 15 wt.% [28] and 20 wt.% [29]. On the other hand, the synthesis of the cellulose-containing PS materials can be performed during a one-step procedure: the monomer emulsion stabilization with cellulose (e.g., regenerated NFC [30] or MFC [31]) is followed by styrene polymerization. However, the cellulose content in the obtained composite materials was low: 2 wt.% [30] and 5 wt.% [31]. Thus, the elaboration of a simple, scalable, one-step synthesis of a cellulose/polymer composite with a high biopolymer content (≥30 wt.%) is of great scientific interest and in high industrial demand.

In this study, we describe the facile way to produce MFC-based composites via a one-step procedure, including the stabilization of the styrene emulsion and its further polymerization. The amount of cellulose in the obtained samples varied from 30 wt.% to 70 wt.%. To the best of our knowledge, the described procedure has been applied to the synthesis of biocomposites containing such a high cellulose content for the first time. The choice of MFC as the reinforcing component was determined by the fortunate combination of its mechanical properties, low coefficient of thermal expansion, and good barrier characteristics. Last but not the least, MFC production includes only a mechanical treatment, which makes it significantly cheaper compared to NFC [29]. Thus, the incorporation of MFC into the polymer matrix can both improve the mechanical properties and reduce the price of the final material [32,33]. Styrene is a good choice for emulsion polymerization thanks to its high reactivity and hydrolytic stability, as well as its comparatively low price [34]. The obtained biocomposite materials can be related to the group of thermoplastics and, thus, can be used in real production. In this work, they were employed for the preparation of sponge hybrid composites that can be applied as sorption materials for the purification and desalination of waters of various origins.

## 2. Materials and Methods

### 2.1. Raw Materials

Styrene monomer (ρ = 0.906 g/cm^3^ at 20 °C, purity ≥ 99.0%, stabilized with 4-tert-butylpyrocatechol), carboxymethylcellulose sodium salt of low viscosity (degree of substitution: 0.65–0.90, Na content 6.5–9.5%), ammonium persulfate ((NH_4_)_2_S_2_O_8_, 97%), and acetone ((CH_3_)_2_O, ≥99.5%) were purchased from Sigma-Aldrich (St. Louis, MO, USA). Softwood sulfate bleached pulp was supplied by Arkhangelsk Pulp and Paper Mill (Arkhangelsk, Russia).

### 2.2. MFC Preparation

To obtain the cellulose microfibrils, we employed the method described previously [35]. A mixture of 2 g of carboxymethylcellulose sodium salt and 500 mL of 5 wt.% of washed wood pulp was stirred vigorously for 1 h at room temperature and then passed through a Masuko supermasscolloider MKCA6-5 (Masuko, Sangyo Co., Ltd., Kawaguchi, Japan). The addition of carboxymethylcellulose salt was necessary to stabilize the cellulose microfibrils and to prevent them from aggregation. After two days of sedimentation of the treated solution, the obtained microfibers were collected.

### 2.3. Synthesis of CMF/PS Composite Fibers

The synthesis of biocomposites based on MFC and PS was reported previously by a few groups of researchers [36,37,38]. In this work, we slightly modified the procedure described earlier [36]. A mixture containing 600 mL of 3 wt.% MFC gel and various amounts of styrene was prepared at different mass ratios of monomer to cellulose (30:70, 50:50, 70:30) in a 1000 mL beaker and ultrasonically treated at 20 kHz, 400 W using the Branson S-450D Digital sonifier (Branson, Danbury, CT, USA) for 10 min. Then, the obtained emulsion was placed in a 1000 mL flask with a condenser. Ammonium persulfate (3 wt.% of styrene monomer mass) was added to the obtained mixture. The mixture was rigorously stirred at 450 rpm and 85 °C until the moment the mixture viscosity increased and its color changed to white. From that moment to the end of the synthesis, the stirring rate was maintained at 610 rpm. The next 100 min of the synthesis the mixture stirring was carried out at the same temperature. To initiate the polymer chain growth, the mixture was cooled down to 65 °C and stirred for the next 40 min. During the last 50 min of the synthesis, the temperature was increased again to 85 °C. The total synthesis time was 190 min. At the end of the synthesis, the obtained sample was left overnight to cool to room temperature in a hermetically sealed vessel, and then it was filtrated on a Buechner funnel, washed with distilled water, and dried at 65 °C until a constant mass was reached. The samples with PS:MFC weight ratios of 70:30, 50:50, 30:70 were denoted as *f*-PS_70_@MFC_30_, *f*-PS_50_@MFC_50_, and *f*-PS_30_@MFC_70_, correspondingly (see Table 1).

### 2.4. Preparation of Sponge Composite Materials

The sponge/porous composite materials were prepared from the well-ground *f*-PS@MFC composite by pressing. A total of 3 g of each *f*-PS@MFC composite was loaded into a 25 mm circular mold, soaked with 15 mL of acetone and pressed by PLG-25 press in manual mode (the maximum pressure was 2 bars). No blowing agent was used. The retrieved samples were dried at 65 °C until a constant mass was reached. The samples obtained from *f*-PS_70_@MFC_30_, *f*-PS_50_@MFC_50_, and *f*-PS_30_@MFC_70_ were denoted as *s*-PS_70_@MFC_30_, *s*-PS_50_@MFC_50_, and *s*-PS_30_@MFC_70_, correspondingly (see Table 1).

### 2.5. Electron Microscopy

The morphologies of the obtained *f*-PS@MFC and *s*-PS@MFC samples were investigated with help of scanning electron microscopy (SEM) using the JEOL JIB 4501 multibeam system (Jeol Ltd., Tokyo, Japan). The samples were fixed to the SEM stubs with help of double-sided carbon tape. In order to avoid undesired overcharge of the samples’ surface and the corresponding artefacts in SEM micrographs, a sputter coating was applied. The 30 nm Au layer was deposited with the help of a Q150R ES Plus ion sputter coater. The SEM micrographs were acquired in the SE mode at 5 kV accelerating voltage in a magnification range of ×1000–×5000.

### 2.6. Fourier-Transform Infrared Spectroscopy (FTIR)

To obtain the corresponding absorption mode, the FTIR spectra of the composite *f*-PS@MFC samples in the 4000–600 cm^−1^ range, a Nicolet iS 10 FTIR Spectrometer with germanium ATR crystal (Thermo Fisher Scientific, Waltham, MA, USA) was employed. The spectral resolution of the device was 8 cm^−1^, and the acquisition time for each spectrum was 14 s. Data recording and processing were performed using OMNIC Thermo Scientific software (version 7.3).

### 2.7. Thermogravimetric Analysis (TGA)

The thermal stability of the obtained fibrous composite materials was investigated by thermogravimetric analysis. The samples (20 mg) were placed in standard corundum crucibles. A Netzsch STA 449 F5 Jupiter synchronous thermal analyzer (NETZSCH Instruments, Selb, Germany) was used to obtain the corresponding thermogravimetric data. The experiment was carried out in a pure nitrogen atmosphere with a heating rate of 10 °C/min in the temperature range of 30 to 600 °C.

### 2.8. Differential Scanning Calorimetry (DSC)

DSC was employed to measure the thermal properties of the obtained fibrous samples. The measurements were carried out with a Netzsch DSC 214 Polyma calorimeter (NETZSCH Instruments, Selb, Germany) in the 20–200 °C temperature range. The heating rate was set to 10 °C/min. The samples (20 mg) were placed in standard aluminum crucibles.

### 2.9. Density, Porosity, and Water Uptake Coefficient of Sponge Composite Materials

The density, porosity, and water uptake coefficient values of the composite porous materials were measured with help of liquid displacement, as was performed for similar materials previously [16,39,40,41,42].

*s*-PS@MFC composites were immersed in distilled water (23 °C) for 5 min until their saturation. The mass of each sponge composite was weighed before and after the immersion in water. After the mass measurement of wet/saturated sample, it was dried at 65 °C until a constant mass was reached and weighted one more time. The total pores volume (*V*_p_) and porosity (µ) were calculated using Formulas (1) and (2):

*V*_p_ = (*M* _wet sample_ − *M* _dry sample_)/ρ_H_2_O_, (1)

µ = 100% × *V*_p_/*V*_s_, (2)

where *M* _wet sample_ is the mass of the wet/saturated sample (g), *M* _wet sample_ is the mass of the sample before immersion (g), *V*_s_ is the volume of the sample before immersion (cm^3^), and ρ_H2O_ is the distilled water density (g/cm^3^).

Additionally, the volumes of water adsorbed by the self-floating *s*-PS@MFC composite materials were investigated. Each sample was placed on the surface of distilled water and left for 24 h. The next step was to measure the mass of the material with the absorbed water (*M*_floating sample_). The water absorption coefficient (*E*) was calculated by Formula (3), similarly to how it was performed earlier [16]:

*E* = (*M* _floating sample_ − *M* _dry sample_)/*M* _dry sample_, (3)

The measurements of *V*_p_ and µ values were provided three times for each composition, while the measurement of *E* was performed once. It should also be underlined that in the calculation of the pores’ total volume and the porosity, the amount of water sorbed by MFC fibers themselves was neglected because of the relatively short contact time of the samples with water.

## 3. Results and Discussion

As mentioned above, to obtain the composite microfibers (*f*-PS@MFC), the procedure described previously for the synthesis of NFC/PS was employed [36]. Significantly longer fibrils of MFC fibrils and, hence, a considerably increased viscosity of the obtained water gel compared to the NFC required a slight adjustment of stirring and temperature regimes, as well as a prolongation of the ultrasonic treatment time (10 min in the present work vs. 3 min in [36]). The use of longer primary ultrasonic treatment made it possible to overcome the inevitable phase separation of initial components and to obtain a stable styrene emulsion owing to a better homogeneous distribution of MFC fibrils in the volume. Moreover, in accordance with previous observations [36], the longer sonication leads to smaller monomer droplets, which considerably accelerates the polymerization rate and PS yield. PS yield was calculated for each sample as the ratio of polymer mass (obtained by subtraction of dry MFC mass from the mass of *f*-PS@MFC composite) to initial monomer mass (see Table 2).

According to Table 2, the PS yield decreases while monomer amount increases. This phenomenon can likely be explained by the high styrene volatility and, hence, greater monomer loss during ultrasonic treatment of the MFC gel/styrene mixture in the beaker. The weight ratios of MFC and PS retrieved from TGA investigations of *f*-PS@MFC composites also supported this assumption. As follows from the data in Table 2, the maximum deviation (5 wt.%) of the gravimetrically determined weight ratio of the MFC and PS from the theoretical one was observed for *f*-PS_70_@MFC_30_, whereas, in case of *f*-PS_30_@MFC_70_, it did not exceed 1 wt.%.

**Figure 1:**
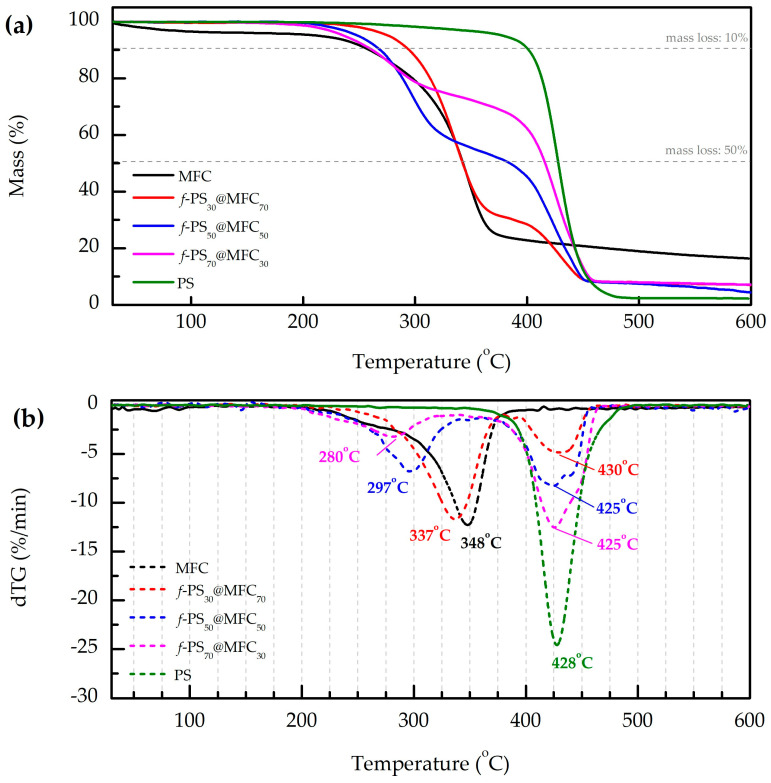
TGA curves of PS, MFC, and *f*-PS@MFC (**a**); the corresponding dTG curves, with the decomposition temperatures underwritten (**b**). The mass of each sample: 20 mg; heating rate: 10 °C/min; atmosphere: N_2_.

The TGA and dTGA curves of *f*-PS@MFC samples are presented in Figure 1.

The shapes of TGA curves of the *f*-PS@MFC samples were found to be similar to those observed for the other cellulose/polymer blends reported previously [43,44]. Thermal decomposition of the *f*-PS@MFC composite occurs in two steps: graphitization of cellulose microfibrils is followed by PS decomposition.

The thermal decomposition of neat MFC and cellulose in *f*-PS@MFC composites took place in the 200–400 °C range. Besides desorption of physically sorbed water, it includes cross-linking of cellulose chains, unzipping of the cellulose chain (transglycosidation), formation of laevoglucosan (which can further decompose releasing volatile products), and decomposition of the dehydrated product yielding char and volatile products [44]. It could be noted that the peak decomposition temperatures of neat cellulose and cellulose in composites varied significantly. The peak decomposition temperature of neat MFC (348 °C) was the maximum, whereas the same parameter of *f*-PS@MFC composites decreased while the cellulose content reduced. Indeed, the same phenomenon was also observed for other cellulose/polymer blends [43,44]. One of the probable interpretations of this phenomenon could be the different kinetics of cellulose thermal decomposition, which depends of the amount of cellulose in the sample.

PS decomposition occurred in the 350–490 °C temperature range. As follows from Figure 1, the peak decomposition temperatures of PS in composite samples were close to the value of neat PS (the maximum deviation did not exceed 3 °C). PS peak decomposition in *f*-PS_30_@MFC_70_ occurred at 430 °C, which is higher than the same value of neat PS (428 °C). In case of the samples with a higher PS content (50 wt.% and 70 wt.%), the opposite was revealed: the peak decomposition temperatures of composites decreased (425 °C for both) compared to the neat PS. Moreover, careful analysis of the PS peaks in the dTG curves of *f*-PS_50_@MFC_50_ and *f*-PS_70_@MFC_30_ revealed shoulders around 440 °C, indicating that the part of polymer decomposed at higher temperature.

Based on the obtained TGA data, we assume that the interaction PS (or/and the products of its thermal decomposition) with cellulose (or/and the products of its thermal decomposition) is the most probable explanation of the observed phenomena. Indeed, the same assumption was made earlier for cellulose/polypropylene compounds [45]: char-forming cellulose led to a slight increase in PP degradation temperature, due to the fact that charcoal promotes the hydrogenation of the unsaturated products and the hydrogenated products evolve at a higher temperature. In case of *f*-PS_30_@MFC_70_, an excess of cellulose led to a shift in peak decomposition temperature towards higher temperatures. In the case of *f*-PS_50_@MFC_50_ and *f*-PS_70_@MFC_30_, a part of PS decomposed at 425 °C without any influence of MFC (owing to lower MFC content), whereas the other part of polymer (presumably the closest to fibril surface) was affected by cellulose and decomposed around 440 °C.

**Figure 2:**
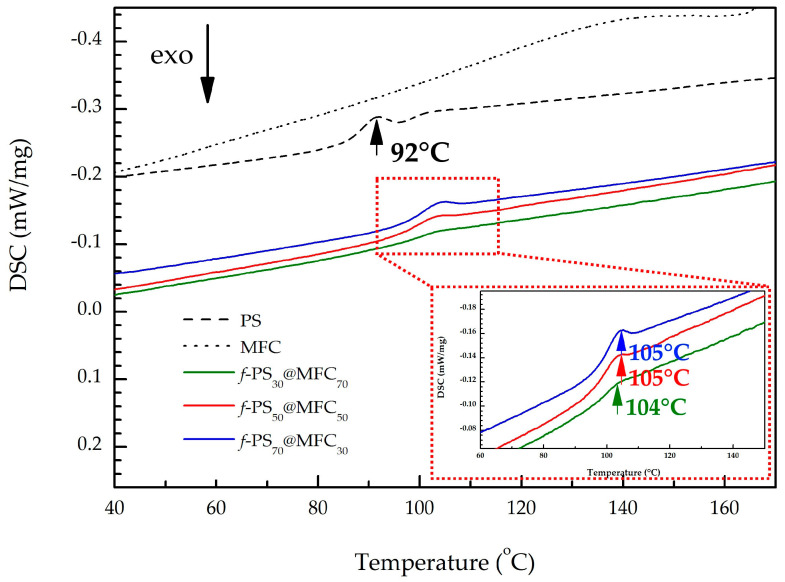
DSC thermograms of PS, MFC, and *f*-PS@MFC composites; the arrows indicate the corresponding temperatures of PS glass transition. The mass of each sample: 20 mg; the heating rate: 10 °C/min.

Figure 2 presents the DSC curves of the *f*-PS@MFC composites and the neat MFC and PS. The glass transition temperatures of PS in the microfibrous composite structures are around 105 °C, which is in agreement with the literature data [31,46,47,48]. It might indicate a possible syndiotactic microstructure of the polymer. The obtained results supported our assumption about the interaction of PS and MFC at elevated temperatures: a slight increase in PS glass transition temperature values of *f*-PS@MFC, compared to that of neat PS, may be the result of the interaction between the PS matrix and the cellulose fibril network [31].

**Figure 3:**
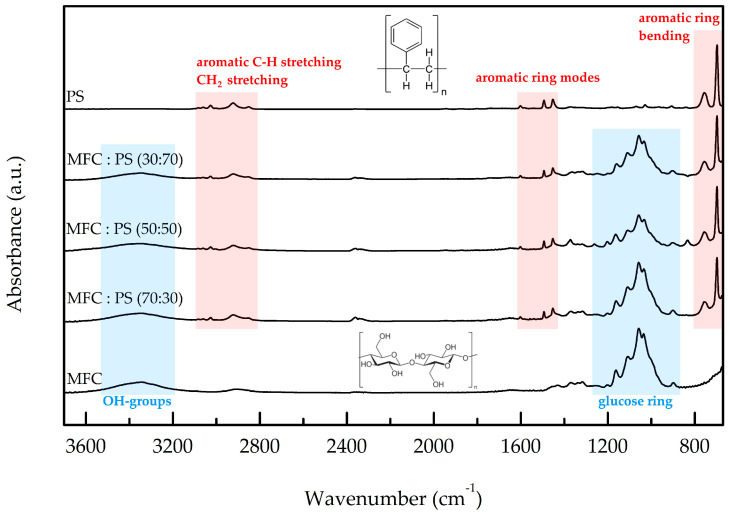
FTIR spectra of PS, MFC, and *f*-PS@MFC composites; the most intense bands referring to the PS and cellulose functional group oscillations are highlighted by red and blue, respectively.

FTIR studies of *f*-PS@MFC composites and pristine MFC and PS are shown in Figure 3. The presented FTIR data are in good agreement with those of NFC/PS reported previously [36]. All spectra of composite materials contain bands in the 3600–3200 cm^−1^ and 1400–900 cm^−1^ wavenumber ranges, referring to glucose OH groups and glucose ring oscillations, respectively, as well as bands in the 1700–1400 cm^−1^ and 3100–2800 cm^−1^ wavenumber ranges, corresponding to aromatic ring modes and aromatic C–H/CH_2_ stretching modes of PS, respectively. All three FTIR spectra of *f*-PS@MFC are identical to each other. Moreover, the comparison of FTIR spectra of composite microfibrils with the spectra of PS and cellulose revealed neither a shift of the corresponding bands nor the emergence of any new features. The latter indicates that there is no chemical interaction/grafting between MFC and PS in *f*-PS@MFC composites at room temperature. This result accords nicely with our previous observation of FTIR spectra of NFC/PS composites [36], and indicates that a physical adsorption of PS particles took place during *f*-PS@MFC synthesis.

**Figure 4:**
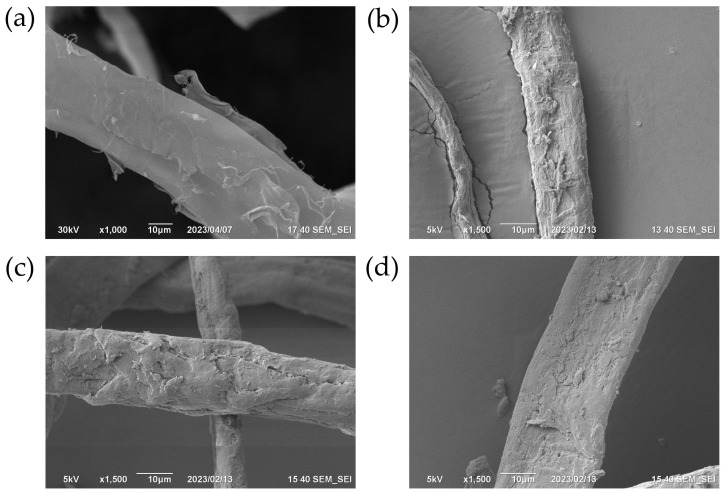
SEM micrographs of MFC (**a**), *f*-PS_30_@MFC_70_ (**b**), *f*-PS_50_@MFC_50_ (**c**), *f*-PS_70_@MFC_30_ (**d**).

The distribution of PS onto the MFC surface was investigated with help of SEM (Figure 4). The pristine MFC is presented as flat fibers with average width of 30 µm. The SEM micrographs of the obtained *f*-PS@MFC samples revealed the dense and uniform distribution of the polymer onto fibrils. It can be noted that the increase in PS mass portion in the composite led to a more uniform and smooth polymer deposition on the MFC. The absence of isolated PS particles might suggest that styrene polymerization process took place exactly onto the MFC fibrils’ surface.

**Figure 5:**
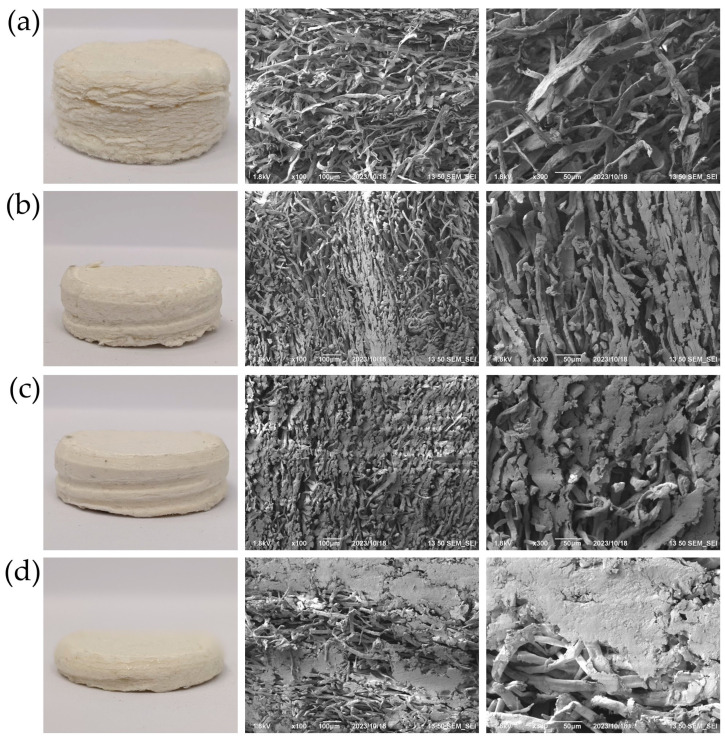
Digital photographs and SEM micrographs (magnification ×100, ×300) of *s*-MFC (**a**), *s*-PS_30_@MFC_70_ (**b**), *s*-PS_50_@MFC_50_ (**c**), *s*-PS_70_@MFC_30_ (**d**).

The *f*-PS@MFC were further employed to prepare the corresponding sponge composite materials, namely the *s*-PS@MFCs (Figure 5). The preparation of the hybrid sponge composite materials was facile, and did not require any special blowing agent, which is obviously advantageous from the environmental point of view. When *f*-PS@MFC was treated by acetone, a small part of PS partially dissolved and worked like a glue, binding the neighbor composite fibrils during the pressing. Thus, the porosity of the final *s*-PS@MFC material can be control by varying the pressing force, as well as the weight ratio of MFC and PS in the *f*-PS@MFC. A preparation of the sponge hybrid composites neither involved a significant modification to *f*-PS@MFC nor an addition of any other components (e.g., a blowing agent). This fact implies that the thermal and phonon properties of *s*-PS@MFC did not significantly vary, and can be considered unchanged compared to initial *f*-PS@MFC; hence, the corresponding investigations (FTIR, TGA, DSC) of sponge hybrid materials were not performed.

The obtained *s*-PS@MFC composites might be a more “green” alternative to the actual water sorption materials; hence, comprehensive investigations of their structure and characteristics such as porosity and water uptake are of great importance [49,50,51,52]. According to Figure 5, the volumes and, hence, the densities of the *s*-PS@MFC samples obtained under the same loading were different. The SEM micrographs in Figure 5 revealed the structure of the obtained hybrid sponge materials. For the sake of comparison, a control sample containing only MFC (*s*-MFC) prepared following the same technology described in Part 2.4 was introduced. The SEM investigation proved our assumption on the role of PS as a glue for the neighbor fibrils: a SEM micrograph of *s*-MFC revealed neat/uncoated cellulose fibrils, while in the composites, the surface coverage of MFCs increases with an increasing PS content.

Table 3 presents the density (ρ), pore volume (*V*_p_), porosity (µ), and water absorption coefficient (*E*). According to the data presented in Table 3, *s*-PS_30_@MFC_70_ possessed the lowest density and highest porosity and water adsorption among all prepared samples during self-floating on water. The water uptake of the *s*-PS_30_@MFC_70_ sample was almost twice as much as the same value for *s*-PS_50_@MFC_50_. It can be explained not only by it having the highest fraction of cellulose in its composition, which can soak some part of water itself, but also by the particularity of the *s*-PS@MFC preparation technology. Evidently, the lower PS amount on the cellulose microfibrils led to a less dense structure, hence a higher porosity level and more active water saturation. The water stored in the pores of such a material was better retained by the hydrophilic MFC, owing to the lower coverage of fibrils by the polymer.

It should be underlined that, despite the rather high weight fraction of the cellulose in *s*-PS_30_@MFC_70_, it preserved its shape and structure after 24 h of floating on water. Such a high wear stability might become a key factor for further industrial applications of the proposed hybrid material. Shape stability of the *s*-MFC was also checked during self-floating in water. After 15 min of observation, the *s*-MFC completely disintegrated, which highlights the crucial role of PS in the composite material composition.

Summing up, the obtained sponge composite materials possess a combination of the required performance features, as well as an improved range of properties compared to the individual components they are made of. The variation in the PS/MFC weight ratio in the *f*-PS@MFC composite and during loading pressure in *s*-PS@MFC preparation allows one to tune not only the porosity/density of the hybrid material, but also its hydrophobicity/hydrophilicity, which might be an advantageous feature compared to the materials currently employed in oil/water mixture separations [50]. The high shape stability of the *s*-PS@MFC material allows it to maintain its structure in a volume of water, even after long time of floating, and to resist numerous damages. MFC fibrils in the material can serve as additional hydrophilic channels for water transport under specific conditions. The PS-based matrix can maintain and retain the shape, so it is possible to re-heat and transform such materials without significantly changing their properties. These features might be valuable in the development of environmentally friendly systems for solar vapor generation [51,53]. In recent decades, there has been a growing interest in cellulose-based thermal insulation materials [54,55,56,57]. Thanks to low the thermal conductivities of both MFC and PS, the resulting hybrid sponge composites might be considered as promising thermal insulation materials in the future. Last but not least, the obtained hybrid composites are characterized by broad availability (hence low cost), negligible environmental impact, and low energy consumption during their creation and processing, making them valuable materials for the sustainable development of the modern industry.

## 4. Conclusions

A facile and low-cost method of synthesis of polystyrene/cellulose fiber composites via Pickering emulsion polymerization was proposed. The obtained samples were comprehensively investigated by several analytical techniques. According to SEM and FTIR investigations, it was discovered that styrene polymerization occurs on cellulose microfibrils; however, there is no chemical bonding between the cellulose and the polystyrene. Nevertheless, DSC and TGA analyses revealed a slight increase in polystyrene glass transformation temperature and peak decomposition temperature compared to the neat polymer.

The obtained composite fibers were further employed to produce sponge/porous hybrid materials. Parameters such as density, pore volume, porosity, and water uptake were determined for the obtained sponge composites. It was found that the sample with the maximum cellulose content (70 wt.%) possessed the minimal density and the highest water adsorption, while preserving its shape after 24 h of floating on water. Thus, the obtained materials can be considered as a promising environmentally friendly alternative to currently employed sorption materials for water purification/desalination.

## Figures and Tables

**Table 1:** The composition of fiber (*f*-) and sponge (*s*-) composite samples.

Sample	PS (wt.%):MFC (wt.%)		
	70:30	50:50	30:70
Composite fibers	*f*-PS_70_@MFC_30_	*f*-PS_50_@MFC_50_	*f*-PS_30_@MFC_70_
Porous composites	*s*-PS_70_@MFC_30_	*s*-PS_50_@MFC_50_	*s*-PS_30_@MFC_70_

**Table 2:** PS yield during the synthesis of *f*-PS@MFC composite microfibers, theoretical mass ratios of MFC and PS, and temperatures for 10% (*T*_10%_mL_), 50% (*T*_50%_mL_) mass loss, residue mass (*M*_r_) at 600 °C, experimental mass ratios of MFC, and PS obtained from TGA data.

	MFC	*f*-MFC_70_/PS_30_	*f*-MFC_50_/PS_50_	*f*-MFC_30_/PS_70_	PS
*T*_10%_mL_, °C	259	295	270	261	401
*T*_50%_mL_, °C	343	342	384	416	428
*M*_r_, %	16.4	7.0	4.4	7.1	2.2
theoretical MFC:PS mass ratio, wt.%	―	70:30	50:50	30:70	―
experimental MFC:PS mass ratio, wt.%	―	71:29	53:47	35:65	―
PS yield, %	―	96.1	94.6	91.3	―

**Table 3:** Density (ρ), pores volume (*V*_p_), porosity (µ), and water absorption coefficient (*E*) of *s*-PS@MFC samples.

Sample	ρ, g/cm^3^	*V*_p_, cm^3^	µ, %	*E*
*s*-PS_30_/MFC_70_	0.29(2)	1.53(2)	22.5(3)	0.25
*s*-PS_50_/MFC_50_	0.40(1)	0.62(1)	9.2(1)	0.13
*s*-PS_70_/MFC_30_	0.51(1)	0.46(1)	6.9(1)	0.16

## Data Availability

All data are presented in this article.
